# Supplementary material for: Metabolomic Profiling of Obese Patients with Altered Intestinal Permeability Undergoing a Very Low-Calorie Ketogenic Diet
Source: Nutrients. 2023 Dec 7;15(24):5026. doi: 10.3390/nu15245026 (PMC10745951; doi:10.3390/nu15245026)
Supplement: Supplementary file 1 [file nutrients-15-05026-s001.zip › nutrients-2727975-supplementary.pdf]

Supplementary Figure S1. Fecal metabolomics DAPC clustering. Discriminant analysis of principal components of volatile organic compounds in obese patients with altered and normal intestinal permeability before (pre) and after (post) VLCKD administration. A) DAPC plot based on discriminant eigen values. B) proportions of successful reassignments (based on the discriminant functions) of individuals to their original cluster. C) DAPC linear discriminants. D) Loading plot reporting the contribution of most impacting var-iables (VOCs) useful in discriminating sample clusters.

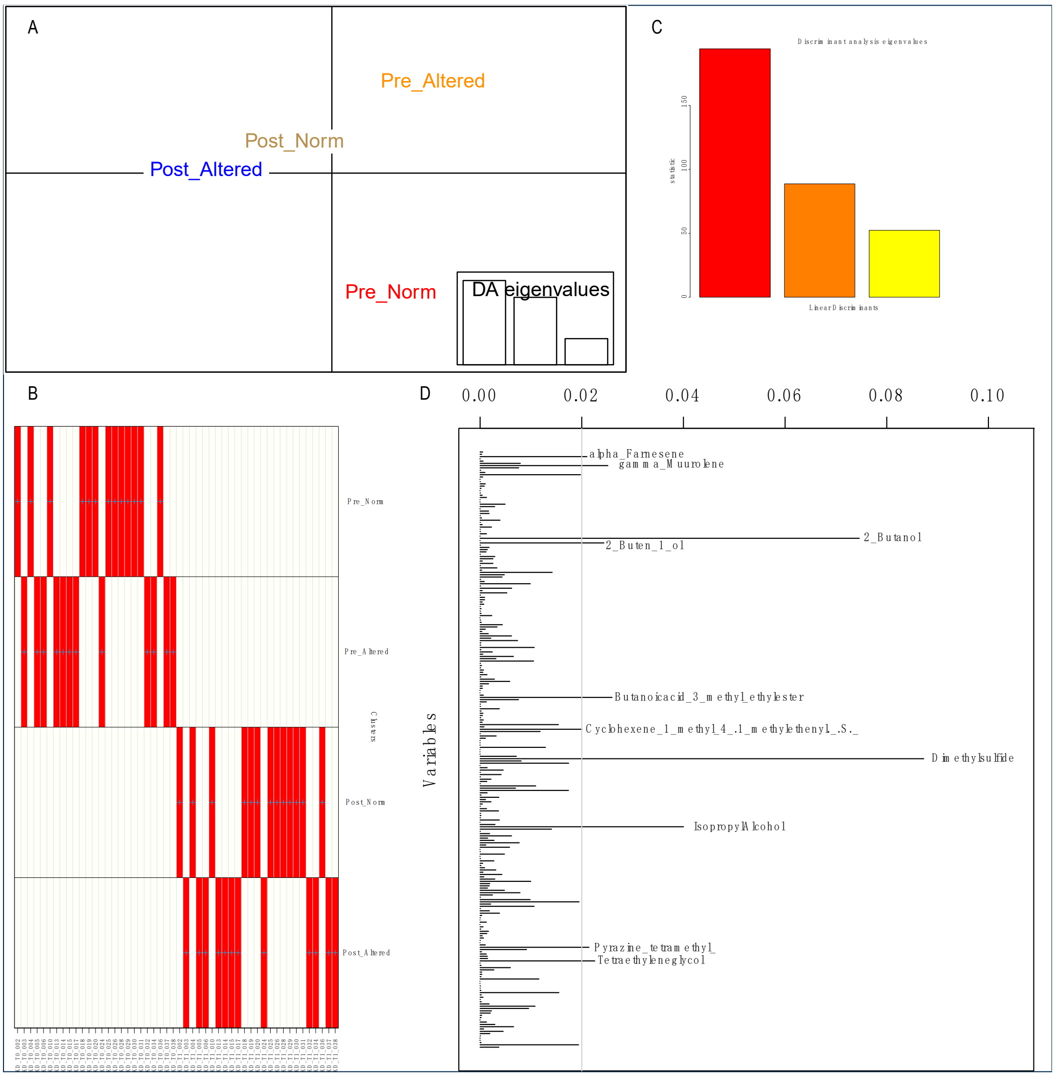

Supplementary Figure S2. Volcano plot of statistically significant fecal VOC fold changes in patients marked by altered intestinal permeability. VOCs in fecal samples from obese patients marked by altered permeability were compared before and after VLCKD administration by meaning of a non-parametric Wilcoxon rank-sum test com-bined with a fold change analysis. Due to the group comparison direction, increased (up-red) and decreased (down-blue) VOC concentrations are relative to altered phenotype after treatment. Under threshold VOCs are reported as grey dots. The table below the volcano plot reports significant VOC fold change as raw values (FC), log2 fold change (log2FC), corrected p-values and the p-value base-10 logarithm.

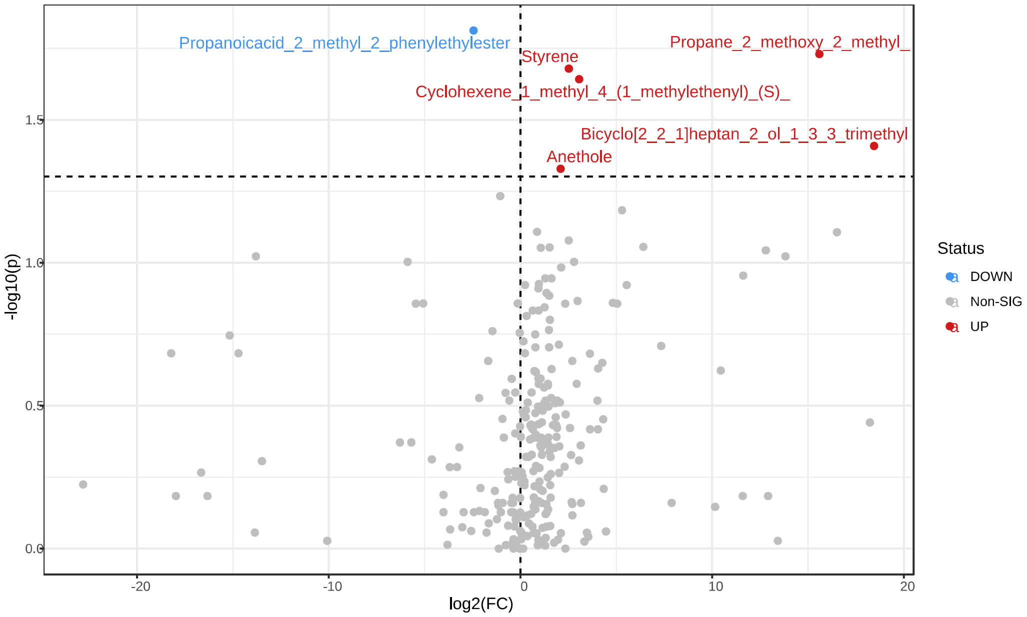

feacal sample VOC	FC	log2(FC)	corrected pval	=-LOG10(p)
Propanoic acid, 2-methyl-, 2-phenylethyl ester	0.18358	-2.4455	0.0154	1.8125
Propane, 2-methoxy-2-methyl-	49393	15.592	0.018635	1.7297
Styrene	5.7559	2.525	0.020954	1.6787
Cyclohexene, 1-methyl-4-(1-methylethenyl)-,(S)-	8.3695	3.0651	0.022805	1.642
Bicyclo[2.2.1]heptan-2-ol, 1,3,3-trimethyl-	357640	18.448	0.039037	1.4085
Anethole	4.2744	2.0957	0.046962	1.3283

Supplementary Figure S3. Volcano plot of statistically significant fecal VOC fold changes in patients marked by normal intestinal permeability. VOCs in fecal samples from obese patients marked by normal intestinal permeability were compared before and after VLCKD administration using a non-parametric Wilcoxon rank-sum test combined with a fold change analysis. Due to the group comparison direction, increased (up-red) and decreased (down-blue) VOC concentrations are relative to normal phenotype after treatment. Under threshold, VOCs are reported as grey dots. The table below the volcano plot reports significant VOC fold change as raw values (FC), log2 fold change (log2FC), corrected p-values and the p-value base-10 logarithm.

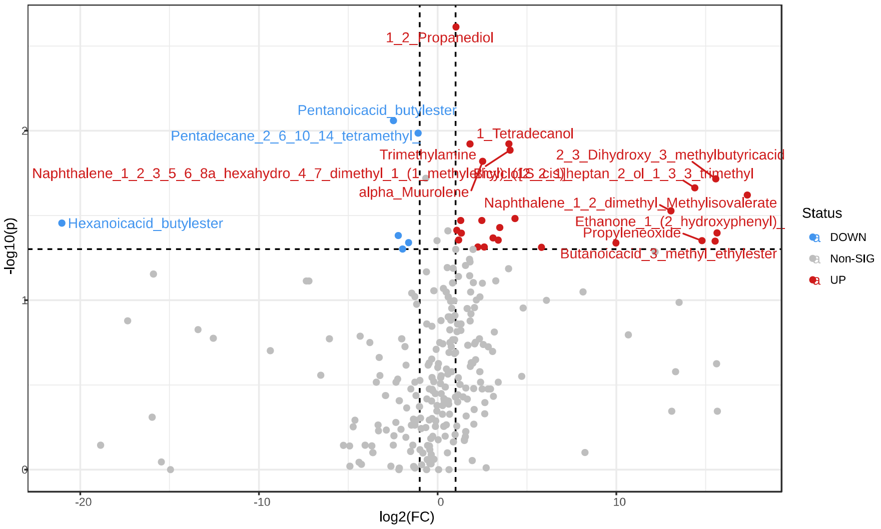

faecal sample VOC	FC	log2(FC)	orrected pva	=-LOG10(p)
1_2_Propanediol	2.0387	1.0276	0.0024389	2.6128
Pentanoicacid_butylester	0.18064	-2.4688	0.0087048	2.0602
Pentadecane_2_6_10_14_tetramethyl_	0.47061	-1.0874	0.010333	1.9858
1_Tetradecanol	15.906	3.9915	0.011949	1.9227
Trimethylamine	3.5047	1.8093	0.011962	1.9222
Naphthalene_1_2_3_5_6_8a_hexahydro_4_7_dimethyl_1_(1_methylethyl)_(1S_cis)_	16.66	4.0583	0.013015	1.8856
alpha_Muurolene	5.7356	2.5199	0.015131	1.8201
2_3_Dihydroxy_3_methylbutyricacid	48369	15.562	0.01921	1.7165
Bicyclo[2_2_1]heptan_2_ol_1_3_3_trimethyl	21453	14.389	0.021736	1.6628
Methylisovalerate	165400	17.336	0.023971	1.6203
Naphthalene_1_2_dimethyl_	8520.1	13.057	0.029693	1.5273
Acetophenone_2_chloro	20.093	4.3287	0.032959	1.482
1_Undecanol	5.549	2.4722	0.033828	1.4707
Caryophyllene	2.4484	1.2918	0.033828	1.4707
Hexanoicacid_butylester	4.68E-07	-21.028	0.035093	1.4548
Tetradecane	11.156	3.4797	0.037287	1.4284
Nonanoicacid	2.1028	1.0723	0.038694	1.4124
Ethanone_1_(2_hydroxyphenyl)_	51023	15.639	0.040061	1.3973
Ethylamine	2.5155	1.3308	0.040205	1.3957
Phenol_3_(1_methylethyl)_methylcarbamate	0.21751	-2.2009	0.041539	1.3815
Cyclopentadecane	8.5933	3.1032	0.042908	1.3675
Hexadecane	2.2513	1.1707	0.044117	1.3554
Menthol	10.499	3.3922	0.044212	1.3545
Propyleneoxide	28377	14.792	0.044536	1.3513
Butanoicacid_3_methyl_ethylester	47354	15.531	0.044776	1.349
Cyclohexene_1_methyl_4_(1_methylethenyl)_(S)_	0.32489	-1.622	0.045759	1.3395
Carbamicacid_methyl_3_methylphenylester	1008.5	9.978	0.045924	1.338
Phenol_3_4_dimethoxy_	6.0973	2.6082	0.048496	1.3143
Heptadecane	4.7572	2.2501	0.048496	1.3143
1_Hexanol_2_ethyl	56.172	5.8118	0.048768	1.3119
Pentanoicacid_propylester	0.25686	-1.961	0.049886	1.302

Supplementary Table S1. SCFA raw concentration average values and relative standard deviations derived from GC-MS target experiment.

SCFA/group	Post_Norm	Pre_Norm	Post_Altered	Pre_Altered
**Acetic acid**	325.83 ± 55.35	320.81 ± 56.81	344.08 ± 79.45	310.5 ± 49.69
**Propanoic acid**	48.43 ± 12.97	51.87 ± 13.58	50.5 ± 13.13	55.86 ± 8.31
**Isobutyric acid**	16.16 ± 11.27	15.14 ± 6.36	16.48 ± 9.24	19.15 ± 9.18
**Butanoic acid**	44.74 ± 20.15	59.46 ± 32.13	41.03 ± 20.73	51.23 ± 21.84
**Isovaleric acid**	11.94 ± 10.49	9.38 ± 3.11	9.32 ± 7	13.69 ± 7.5

Supplementary Table S2. Biochemical parameters expressed as mean and standard deviations.

	Post_Norm	Pre_Norm	Post_Altered	Pre_Altered
**%Lac**	0.239 ± 0.07	0.235 ± 0.13	0.498 ± 0.14	0.292 ± 0.15
**%Man**	11.436 ± 3.05	15.311 ± 6.52	13.814 ± 3.08	11.499 ± 2.65
**Lac to Man**	0.021 ± 0.003	0.015 ± 0.006	0.021 ± 0.005	0.043 ± 0.01
**Fecal Zonulin**	263.928 ± 174.36	255 ± 97.88	221.545 ± 108.48	216.909 ± 135.45
**Serum Zonulin**	44.928 ± 22.58	33 ± 10.49	32.545 ± 11.45	37.545 ± 20.44
**I-FAB**	2.389 ± 1.11	2.11 ± 1.66	2.347 ± 1.04	2.391 ± 1.22
**DAO**	54.214 ± 7.86	55.071 ± 6.74	56.209 ± 4.94	55.39 ± 6.55
**IL-6**	6.117 ± 2.04	6.735 ± 2.93	5.957 ± 2.6	5.818 ± 2.63
**IL-8**	4.685 ± 1.91	5.23 ± 2.2	5.521 ± 1.21	4.897 ± 1.23
**IL-10**	3.292 ± 1.42	3.212 ± 1.03	3.251 ± 0.82	2.52 ± 0.79
**TNF-α**	6.185 ± 2.18	6.894 ± 3.16	5.257 ± 1.33	5.873 ± 1.17
**LPS**	0.044 ± 0.05	0.031 ± 0.03	0.055 ± 0.07	0.023 ± 0.02
**Vitamin D**	26.642 ± 14.53	22.357 ± 10.08	24.4 ± 5.96	20.7 ± 6.48
